# Supplementary material for: Landscape diversity and local temperature, but not climate, affect arthropod predation among habitat types
Source: PLoS One. 2022 Apr 29;17(4):e0264881. doi: 10.1371/journal.pone.0264881 (PMC9053821; doi:10.1371/journal.pone.0264881)
Supplement: S1 Table — (presence-absence of attack from a plot, binomial generalized linear mixed model) including local mean temperature during artificial caterpillar exposure as predictor compared to an empty model (null, null model). Bold font highlights the best model based on ΔAICc < 2 and parsimony. (PDF) [file pone.0264881.s001.pdf]

S1 Table. Model output on the probability of arthropod attack on plot level (presence-absence of attack from a plot, binomial generalized linear mixed model) including local mean temperature during artificial caterpillar exposure as predictor compared to an empty model (null, null model). Bold font highlights the best model based on ΔAICc < 2 and parsimony.

Model	Estimate	df	AICc	ΔAICc
**incl. mean temperature**	1.58	3	89.4	0.00
(null)		2	106.5	17.02
