# Supplementary material for: Landscape diversity and local temperature, but not climate, affect arthropod predation among habitat types
Source: PLoS One. 2022 Apr 29;17(4):e0264881. doi: 10.1371/journal.pone.0264881 (PMC9053821; doi:10.1371/journal.pone.0264881)
Supplement: S2 Table — Included in multimodel averaging on arthropod predation rate models based on 113 study sites. Significant correlations based on α = 0.05 are indicated as following: P < 0.05*, P < 0.01**, P < 0.001***. (PDF) [file pone.0264881.s002.pdf]

S2 Table. Predictor variable details and Pearson's correlation coefficients included in multimodel averaging on arthropod predation rate models based on 113 study sites. Significant correlations based on α = 0.05 are indicated as following: $P < 0.05$*, $P < 0.01$**, $P < 0.001$***.

Scale [km]	Predictor	Min	1st Qu	Median	Mean	3rd Qu	Max	Pearson's correlation coefficients SpecNum	Temp	RH	MAT
	SpecNum	4	20	27	25.7	31	50				
	Temp	2.9	6.6	8.6	8.7	10.3	19.7	0.32***			
	RH	53.6	70.5	79.8	77.7	84.8	100.0	-0.28**	-0.24*		
	MAT	4.5	7.8	8.4	8.3	8.9	10.0	0.33***	0.59***	-0.51***	
0.5	LandDiv	0.0	0.5	0.9	0.8	1.1	1.4	0.13	-0.07	0.05	0.02
1.0	LandDiv	0.0	0.7	0.9	0.9	1.2	1.6	0.09	-0.13	0.08	0.00
1.5	LandDiv	0.0	0.8	1.1	1.0	1.2	1.6	0.06	-0.09	0.07	0.06
2.0	LandDiv	0.1	0.9	1.1	1.0	1.2	1.6	0.03	-0.07	0.09	0.07
2.5	LandDiv	0.1	0.9	1.1	1.1	1.2	1.6	-0.00	-0.06	0.11	0.07
3.0	LandDiv	0.1	1.0	1.1	1.1	1.3	1.6	-0.03	-0.06	0.14	0.03

Min: Minimum, Max.: Maximum, Qu: Quantile, SpecNum: plant species richness, Temp or RH: local mean temperature [°C] or mean relative humidity [%] during artificial caterpillar exposure, LandDiv: landscape diversity (Shannon index), MAT: Multi-annual mean temperature
