# Supplementary material for: Landscape diversity and local temperature, but not climate, affect arthropod predation among habitat types
Source: PLoS One. 2022 Apr 29;17(4):e0264881. doi: 10.1371/journal.pone.0264881 (PMC9053821; doi:10.1371/journal.pone.0264881)
Supplement: S3 Table — (zero-inflated binomial generalized linear mixed model) with different parametrization (best: best model based on ΔAICc < 2 and parsimony; ΔAICc < 4; full: model containing all candidate predictors; null: null model with or without temperature as zero-inflation term) at the best spatial scale identified by multimodel averaging (2-km scale). Best model is highlighted in bold font. (PDF) [file pone.0264881.s003.pdf]

S3 Table. Model output of arthropod predation rate models (zero-inflated binomial generalized linear mixed model) with different parametrization (best: best model based on ΔAICc < 2 and parsimony; ΔAICc < 4; full: model containing all candidate predictors; null: null model with or without temperature as zero-inflation term) at the best spatial scale identified by multimodel averaging (2-km scale). Best model is highlighted in bold font.

	Candidate predictors												
Model	Habitat	Spec Num	Temp	RH	Temp(zi)	LandDiv	MAT	df	AICc	ΔAICc	Pseudo R^2_m	Pseudo R^2_c	VIF_{max}
**best**					**-2.44**	**0.59**		5	591.4	1.81	0.08	0.25	-
ΔAICc<4				-0.27	-2.40	0.58		6	589.6	0.00	0.09	0.23	1.00
ΔAICc<4		-0.06		-0.28	-2.41	0.59		7	591.3	1.73	0.09	0.24	1.05
ΔAICc<4			0.10	-0.25	-2.39	0.58		7	591.4	1.78	0.09	0.23	1.05
ΔAICc<4				-0.24	-2.39	0.57	0.07	7	591.7	2.11	0.09	0.23	1.18
ΔAICc<4			0.15		-2.43	0.60		6	592.5	2.90	0.08	0.25	1.01
ΔAICc<4					-2.41	0.58	0.16	6	592.5	2.91	0.09	0.24	1.01
ΔAICc<4		-0.07	0.12	-0.26	-2.41	0.60		8	592.9	3.32	0.09	0.24	1.10
ΔAICc<4		-0.04			-2.46	0.60		6	593.4	3.77	0.08	0.26	1.03
ΔAICc<4		-0.06		-0.26	-2.40	0.59	0.07	8	593.4	3.84	0.09	0.24	1.19
full	+	-0.08	0.13	-0.27	-2.42	0.62	0.01	12	601.8	12.18	0.10	0.24	1.55
null+ Temp(zi)					-2.07			4	625.1	35.48	0.00	0.14	-
null								3	638.7	49.15	0.00	0.16	-

SpecNum: plant species richness, Temp or RH: local mean temperature [°C] or mean relative humidity [%] during artificial caterpillar exposure, LandDiv: landscape diversity (Shannon index), MAT: Multi-annual mean temperature [°C], zi: included as zero-inflation term, df: Degrees of freedom, AICc: Akaike's information criterion corrected for small sample size, ΔAICc: Difference in AICc relative to minimum value, Pseudo R^2: marginal (only fixed effects) and conditional (fixed + random effects) Nakagawa R^2 values, VIF_{max}: Highest variance inflation factor
