# Supplementary material for: Landscape diversity and local temperature, but not climate, affect arthropod predation among habitat types
Source: PLoS One. 2022 Apr 29;17(4):e0264881. doi: 10.1371/journal.pone.0264881 (PMC9053821; doi:10.1371/journal.pone.0264881)
Supplement: S4 Table — (zero-inflated binomial generalized linear mixed model) at the best spatial scales identified by multimodel averaging (2-km scale). Interaction terms are added to the original best model and to null models containing only temperature during exposure as zero-inflation term. Asterisks between candidate predictors indicate that both main effects and their interaction term is included. Best model parametrization is derived based on ΔAICc < 2 and parsimony. Best models are highlighted in bold font. (PDF) [file pone.0264881.s004.pdf]

S4 Table. Model output of arthropod predation rate models including interactive effects with habitat type (zero-inflated binomial generalized linear mixed model) at the best spatial scales identified by multimodel averaging (2-km scale). Interaction terms are added to the original best model and to null models containing only temperature during exposure as zero-inflation term. Asterisks between candidate predictors indicate that both main effects and their interaction term is included. Best model parametrization is derived based on ΔAICc < 2 and parsimony. Best models are highlighted in bold font.

Interaction term<br>Model	Candidate predictors	df	AICc	ΔAICc	Pseudo R^2_m	Pseudo R^2_c	VIF_{max}
*Habitat*SpecNum*							
original.best + interaction	Habitat*SpecNum + LandDiv + Temp(zi)	12	599.8	8.45	0.09	0.27	13.98
interaction	Habitat*SpecNum + Temp(zi)	11	631.4	40.01	0.01	0.15	11.08
**best**	**LandDiv + Temp(zi)**	5	591.4	0.00	0.08	0.25	-
*Habitat*Temp*							
original.best + interaction	Habitat*Temp + LandDiv + Temp(zi)	12	597.2	5.83	0.10	0.25	3.92
interaction	Habitat*Temp. + Temp(zi)	11	616.1	24.66	0.04	0.15	2.87
**best**	**LandDiv + Temp(zi)**	5	591.4	0.00	0.08	0.25	-
*Habitat*RH*							
original.best + interaction	Habitat*RH + LandDiv + Temp(zi)	12	594.6	3.22	0.11	0.26	2.16
interaction	Habitat*RH. + Temp(zi)	11	632.6	41.23	0.02	0.14	2.09
**best**	**LandDiv + Temp(zi)**	5	591.4	0.00	0.08	0.25	-
*Habitat*LandDiv*							
original.best + interaction = interaction	Habitat*LandDiv + Temp(zi)	11	594.7	3.33	0.12	0.29	4.70
**best**	**LandDiv + Temp(zi)**	5	591.4	0.00	0.08	0.25	-
*Habitat*MAT*							
original.best + interaction	Habitat*MAT + LandDiv + Temp(zi)	12	591.0	0.00	0.10	0.24	4.67
interaction	Habitat*MAT + Temp(zi)	11	622.1	31.06	0.04	0.14	5.22
**best**	**LandDiv + Temp(zi)**	5	591.4	0.36	0.08	0.25	-

Df: Degrees of freedom, AICc: Akaike's information criterion corrected for small sample size, ΔAICc: Difference in AICc relative to minimum value, Pseudo R^2: marginal (only fixed effects) and conditional (fixed + random effects) Nakagawa R^2 values, VIF_{max}: Highest variance inflation factor, SpecNum: plant species richness, Temp or RH: local mean temperature [°C] or mean relative humidity [%] during artificial caterpillar exposure, LandDiv: landscape diversity (Shannon index), MAT: Multi-annual mean temperature, zi: included as zero-inflation term
