# Supplementary material for: Landscape diversity and local temperature, but not climate, affect arthropod predation among habitat types
Source: PLoS One. 2022 Apr 29;17(4):e0264881. doi: 10.1371/journal.pone.0264881 (PMC9053821; doi:10.1371/journal.pone.0264881)
Supplement: S1 Fig — Squares indicate locations of study regions. Different colours represent the 15 combinations of climate zones (1–5: multi-annual mean temperature from 1981–2010; < 7°C, in 0.5°C steps to 9°C, > 9°C) and regional land-use types (nature = near-natural, agriculture and urban), in four replicates. Regional land-use types were defined as near-natural when > 85% of the region were covered by near-natural vegetation with a minimum of 50% forest, as agriculture when > 40% were covered by arable land and managed grassland, and as urban when > 14% were covered by housing, industry and traffic infrastructure. The land cover map, to the right, shows six main land use types (different colours), three plot locations marked by “x” within the dominating land use types of the region, and 1-km “buffer zones” around the plots. (PDF) [file pone.0264881.s005.pdf]

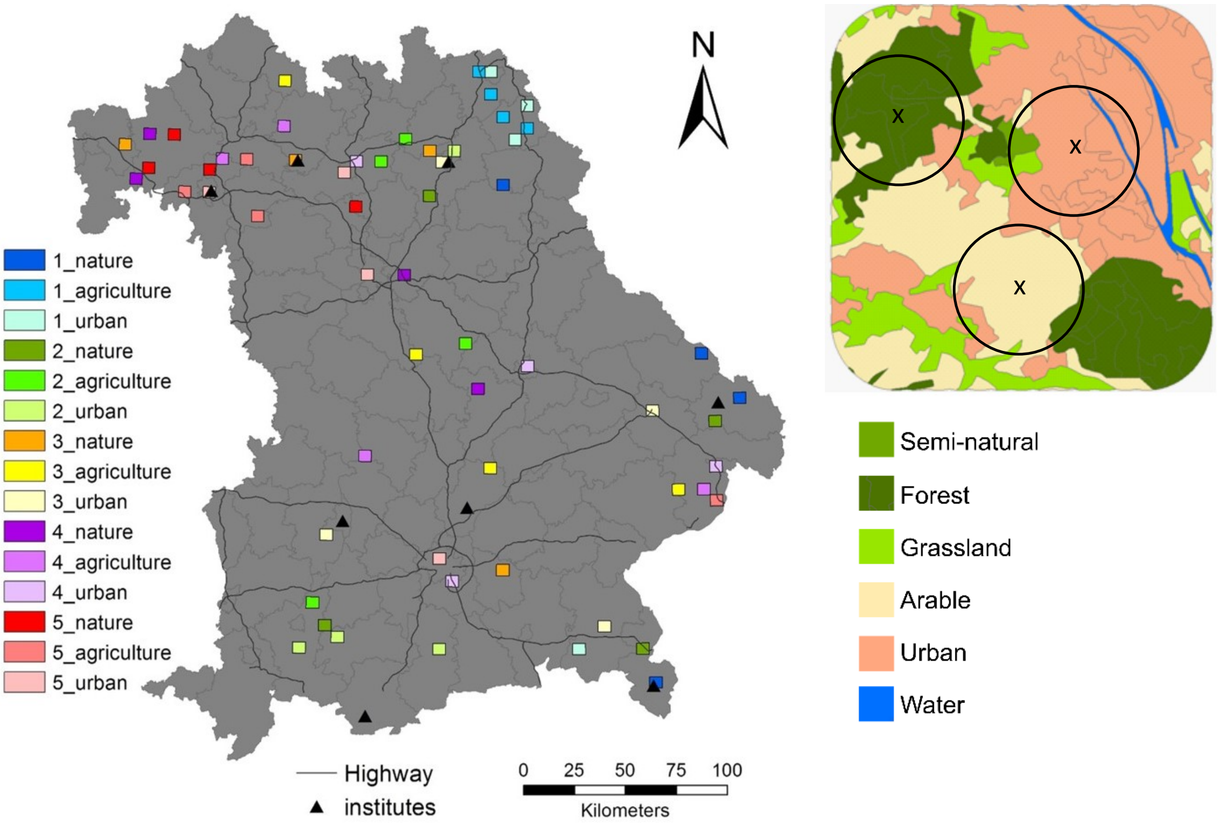

S1 Fig. Maps showing study regions within Bavaria, Germany (left), and plots within an example region (right). Squares indicate locations of study regions. Different colours represent the 15 combinations of climate zones (1–5: multi-annual mean temperature from 1981–2010; < 7 °C, in 0.5 °C steps to 9°C, > 9°C) and regional land-use types (nature = near-natural, agriculture and urban), in four replicates. Regional land-use types were defined as near-natural when > 85% of the region were covered by near-natural vegetation with a minimum of 50% forest, as agriculture when > 40% were covered by arable land and managed grassland, and as urban when > 14% were covered by housing, industry and traffic infrastructure. The land cover map, to the right, shows six main land use types (different colours), three plot locations marked by “x” within the dominating land use types of the region, and 1-km “buffer zones” around the plots.
