# Supplementary material for: Landscape diversity and local temperature, but not climate, affect arthropod predation among habitat types
Source: PLoS One. 2022 Apr 29;17(4):e0264881. doi: 10.1371/journal.pone.0264881 (PMC9053821; doi:10.1371/journal.pone.0264881)
Supplement: S2 Fig — Dots indicate values per plot; overlapping dots appear darker. The dashed grey line presents a hypothetically perfect correlation (r = 1) and the solid black line, the observed correlation based on α = 0.05, P < 0.001***. (PDF) [file pone.0264881.s006.pdf]

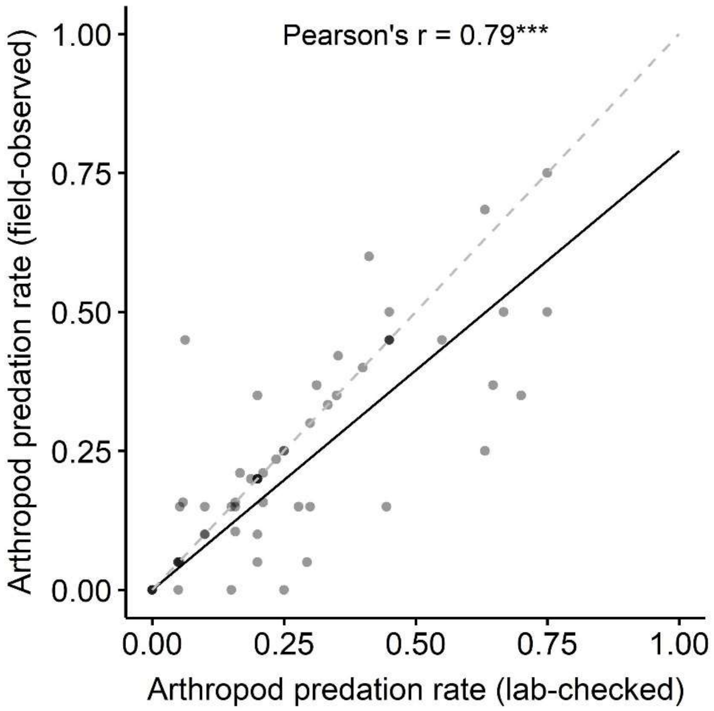

S2 Fig. Pearson correlation between arthropod predation rates assessed in the field and in the lab. Dots indicate values per plot; overlapping dots appear darker. The dashed grey line presents a hypothetically perfect correlation (r = 1) and the solid black line, the observed correlation based on α = 0.05, P < 0.001***.
