# Supplementary material for: Landscape diversity and local temperature, but not climate, affect arthropod predation among habitat types
Source: PLoS One. 2022 Apr 29;17(4):e0264881. doi: 10.1371/journal.pone.0264881 (PMC9053821; doi:10.1371/journal.pone.0264881)
Supplement: S3 Fig — Dots indicate values per plot; overlapping dots appear darker. Asterisks highlight significance levels of P < 0.05* and P < 0.01**. Letters indicate significant differences between habitat types based on Bonferroni-corrected pairwise comparisons using Wilcoxon rank sum test. (PDF) [file pone.0264881.s007.pdf]

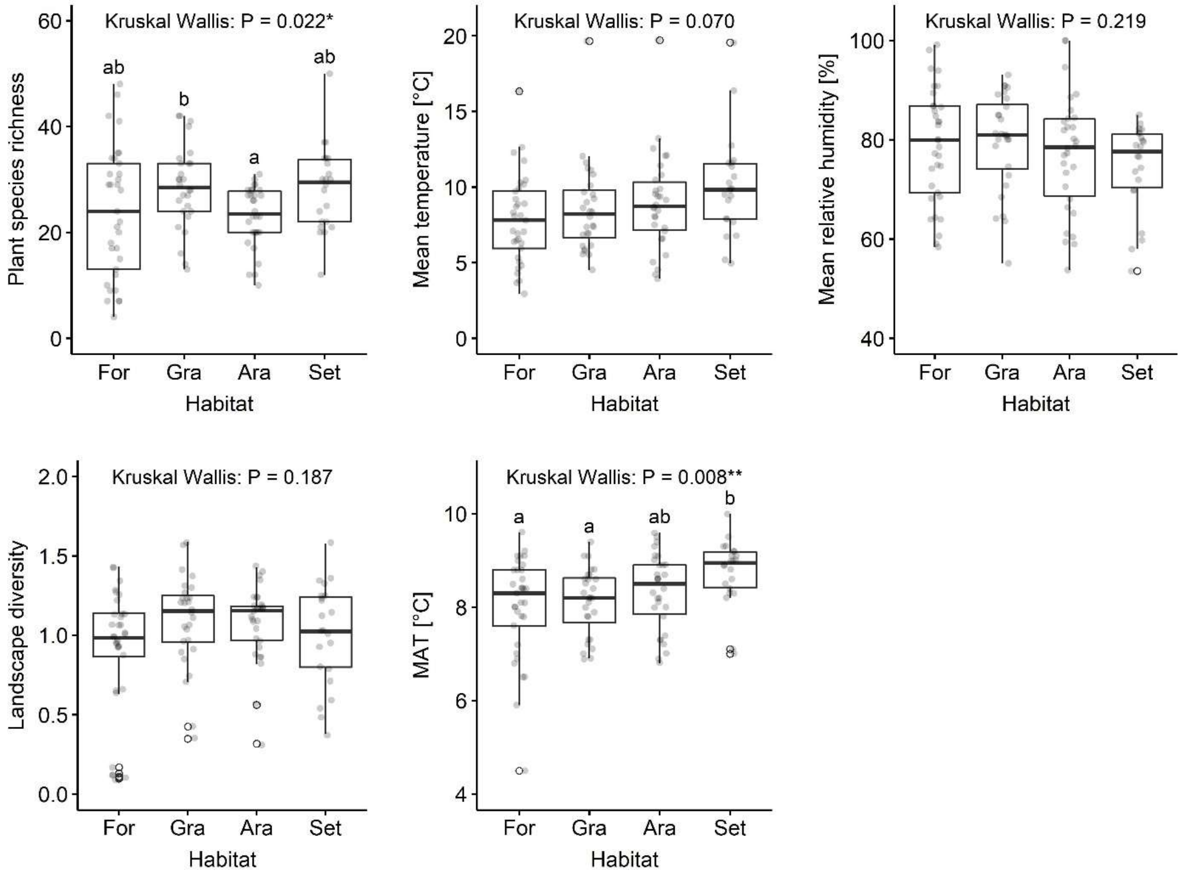

S3 Fig. Relationship between habitat type and other candidate predictors of arthropod predation rates. Dots indicate values per plot; overlapping dots appear darker. Asterisks highlight significance levels of P < 0.05* and P < 0.01**. Letters indicate significant differences between habitat types based on Bonferroni-corrected pairwise comparisons using Wilcoxon rank sum test.
