# Supplementary material for: Landscape diversity and local temperature, but not climate, affect arthropod predation among habitat types
Source: PLoS One. 2022 Apr 29;17(4):e0264881. doi: 10.1371/journal.pone.0264881 (PMC9053821; doi:10.1371/journal.pone.0264881)
Supplement: Attachment — Submitted filename: Response-to-reviewers.docx [file pone.0264881.s008.docx]

# RESPONSE TO EDITOR

Dear Prof. Eve Veromann,

Thank you for the quick handling and the opportunity to submit a revised version of our manuscript. We appreciated the comments by the two reviewers, they helped to improve the readability of the manuscript to the broad audience of PLOS ONE. We have incorporated the suggested changes (see copy of the manuscript with tracked changes) and provide a point-by-point response to the reviewers’ comments (see below). We added information regarding required permits in the methods section and have updated our data availability statement to reflect our intention to make the R code available as well upon acceptance (via Zenodo), in addition to the data. We have also made adjustments to the formatting and the references to meet PLOS ONE's style requirements.

Yours sincerely, also on behalf of the co-authors,

Ute Fricke

# RESPONSE TO REVIEWER 1

Reviewer #1: Dear Editor Prof. Eve Veromann,

Dear Authors,

please find my comments on the paper “Landscape diversity and local temperature, but not climate, affect arthropod predation among habitat types”

The authors showed a very well done and intriguing piece of research about arthropod predation in temperate areas. They correctly pointed out the innovation their research fulfilled: a rare piece of research which study, citing the paper, “the combined effects of local habitat type, plant species richness, weather, landscape diversity and multi-annual mean temperature on arthropod predation”. These data, even if limited in the continental European region, are very interesting to understand the amount of predation rates we nowadays can expect in a vast portion of Europe, and their results, as they pointed out, can help to understand what the potential is to increase these rates through landscape management.
In the introduction, the authors reported concisely, but logically and exhaustive, the context of the research on arthropod predation, as well as open research question and we can clearly read the logic behind the experiment layout we’re going to read in the M&M section.
They measured arthropod predation in an impressive series of plot (147 out of 179 plots, which they reduce, correctly, to 113 after a critic revision of their dataset) in the Bavaria region, Germany. The methodologies they uses are correct, as well as site selection criteria are almost clear and reasonable.
Data analysis have been done correctly, deeply, and using modern techniques, programs, and rationale. I would like to compliment with the authors, because this is one of the best examples of the use of statistics to achieve comprehensive results I recently found.
The authors analysed the results in a logic way. They read their results formulating clear hypotheses fond on recent and appropriate research, but at the same time they were very honest about the limits of their findings. In addition, they correctly criticized the validity of their finding because of plasticine predation limits (like any other experiment of this kind in field, I would say).

Thus, my suggestion to Prof. Veromann is to accept the paper with (very) minor revision. Here I report lineby line comments.

Many thanks for your kind words and the very positive feedback on our manuscript.

Line 90. I’m missing here the amount (and rational) of climate zones. I suspect they’re 5 climatic zones X 3 landscape-scale land use types = 15 combinations. Are the climatic zones from the reference n° 30? I tried to check it, but it is not so user-friendly. Please explain how, of the source of climatic zones.

Thank you for pointing this out. Climate zones were defined as categories of multi-annual mean temperature. We added more info at the respective position in the manuscript (L95, revised version with tracked changes).

# RESPONSE TO REVIEWER 2

Line numbers in our response refer to the **revised version of the manuscript with tracked changes.**

Reviewer #2: In this manuscript, Fricke and colleagues used an innovative multi-scale sampling design to disentangle the effects of multiple drivers on arthropod predation rate. Specifically, they tested how arthropod predation rate varied in contrasting habitats across climate and land-use gradients. I am very enthusiastic about the novel sampling design adopted by the authors, although some additional information describing in more detail the selection and the characteristics of the selected ‘regions’ and ‘plots’ would be beneficial (see specific comments below). The manuscript is well written and a pleasure to read. This allowed the manuscript to be quickly and easily understood. Although the results are in any case interesting, I would have expected, especially considering the sampling design, a major role of climate and a possible interactive effect between climate and landscape on this important function. I am wondering if this could be related to the sampling approach (just a unique sampling date) adopted by the authors. Anyway, this work is certainly of interest, but there are still several minor questions that the authors should address. Hope the authors find my comments helpful.

Thank you for the positive feedback and helpful suggestions. As requested, we added more information on study site selection (L93–111, S1_Fig new version). With regard to the absence of a multi-annual mean temperature effect, our initial expectation also was different. We can’t change the results – besides, results opposing the expectation can be particularly interesting – and discussed this finding in the respective section. We don’t expect that the absence of a multi-annual mean temperature effect results from sampling only once, as our extensive design compensates for this. However, as discussed, our results are limited by the methods used and finding out which ecological filters are applied by the artificial caterpillar method will be an interesting research question for future studies.

SPECIFIC COMMENTS:

L59 It is not clear what you mean by ‘correlation of the latter’.

We rephrased this sentence (L61–63).

L81 I would spend a few more words why you used this method to measure arthropod predation rate. It’s clear reading the Method section, but I think it is also important to introduce it earlier.

Done (L84–85)

L89-91 The sampling design seems very interesting, but I would like to know something more how the selection was done and what are the characteristics of each combination (e.g. climate, landscape, etc.). Also a figure representing the sampling design might be useful.

Done. We added more detail in the text (L93–99) and a figure to the supplement illustrating the study design (S1 Fig, in the revised version)

L93 How did you define typical for the respective region? Did you use a specific threshold?

We replaced the word “typical” by “dominating” and added more information in the text (L99–105). In brief, we selected habitat types by proportion of land-use types in the region.

L94 Also in forests?

Yes, plots were standardized to open herbaceous vegetation. We added examples, what this means with respect to different habitat types, e.g. forest glades (L105–106).

L115 Just one sampling per plots? Right?

Yes. We added “once per plot” (L130)

L119-121 I understand the reasoning of that, but there is a risk that the possible habitat effect can be masked in this specific case (predation rate measured using green artificial caterpillars and mostly determined by ground-dwelling predators). The herbaceous vegetation, regardless of the habitat where is embedded, has the same effect.

We agree that it would have been interesting to compare predation rates on open herbaceous vegetation and inside adjoining habitat type in case of forests and arable fields, but these data were not obtained and it would not have been feasible due to time constraints. However, differences in predation rates inside forests or fields compared to open herbaceous vegetation could also occur due to differences in artificial caterpillar exposure, e.g. placed on ploughed ground, on mosses or on a thick layer of leaf litter. In this study, we standardized artificial caterpillar to ground level in open herbaceous vegetation, which allows us to capture differences in predation rates along climate and land use gradients due to changes in predator community composition, predator behavior or both. Furthermore, we assume that the habitat types adjacent to an experimental strip affects the predator community composition on the strip. We argue that the absence of a habitat type effect is unlikely to result from the absence of an effect from the adjoining habitat types on the predator community, but rather from differences in permeability of the habitat type borders introducing large variation in predation rates within habitat types (also see discussion).

L 148 Would it be possible to make the code used open?

Yes, we intend to make the R code available on Zenodo upon acceptance and adapted the data availability statement accordingly.

L 258-261 In my opinion, what you are observing here is not a habitat effect per se, as you sampled an open herbaceous vegetation in all habitats. The microhabitat conditions (herbaceous vegetation) are more important than the habitat in se. This is might particular true for ground-dwelling predators.

We agree that microhabitat conditions may affect predators, their activity and consequently predation rate. Nonetheless, we think that our comparison between habitat types is also interesting and justified. We assume that research on open herbaceous vegetation adjoining different habitat types (e.g. forest glade, field margin, settlement) captures differences in arthropod community composition, e.g. due to dispersal of species between adjoining habitat types and differences in habitat amounts available to the community. That the resulting differences in arthropod communities persist even despite sampling on standardized strips of herbaceous vegetation was shown in our previous publication, where we observed differences in flying insect biomass and richness among our habitat types (Uhler et al 2021).

Uhler, J., Redlich, S., Zhang, J., Hothorn, T., Tobisch, C., Ewald, J., … Müller, J. (2021). Relationship of insect biomass and richness with land use along a climate gradient. Nature Communications, 12 (5946). doi: 10.1038/s41467-021-26181-3

L 283 This is true Just in part according to your results and what you wrote below.

We added “partially” to weaken the statement (L298).

L305 What do you mean with ‘long-term temperature’? Not clear to me!

“Long-term temperature” was intended to provide another wording for MAT. However, to avoid confusion, we removed “through long-term temperature” as it provided redundant information (L321).

L344 Why only for agricultural ecosystems?

We added a comma before the “and” to make clear the “agricultural ecosystem” only refers to “natural pest control” (L360).
